# Pathogenic Impact of α-Synuclein Phosphorylation and Its Kinases in α-Synucleinopathies

**DOI:** 10.3390/ijms23116216

**Published:** 2022-06-01

**Authors:** Ichiro Kawahata, David I. Finkelstein, Kohji Fukunaga

**Affiliations:** 1 Department of CNS Drug Innovation, Graduate School of Pharmaceutical Sciences, Tohoku University, Sendai 980-8578, Japan; 2 Florey Institute of Neuroscience and Mental Health, University of Melbourne, Melbourne, VIC 3010, Australia; david.finkelstein@florey.edu.au; 3 BRI Pharma Inc., Sendai 982-0804, Japan

**Keywords:** α-synuclein, phosphorylation, Parkinson’s disease, dementia with Lewy bodies, multiple system atrophy, α-synucleinopathy

## Abstract

α-Synuclein is a protein with a molecular weight of 14.5 kDa and consists of 140 amino acids encoded by the *SNCA* gene. Missense mutations and gene duplications in the *SNCA* gene cause hereditary Parkinson’s disease. Highly phosphorylated and abnormally aggregated α-synuclein is a major component of Lewy bodies found in neuronal cells of patients with sporadic Parkinson’s disease, dementia with Lewy bodies, and glial cytoplasmic inclusion bodies in oligodendrocytes with multiple system atrophy. Aggregated α-synuclein is cytotoxic and plays a central role in the pathogenesis of the above-mentioned synucleinopathies. In a healthy brain, most α-synuclein is unphosphorylated; however, more than 90% of abnormally aggregated α-synuclein in Lewy bodies of patients with Parkinson’s disease is phosphorylated at Ser129, which is presumed to be of pathological significance. Several kinases catalyze Ser129 phosphorylation, but the role of phosphorylation enzymes in disease pathogenesis and their relationship to cellular toxicity from phosphorylation are not fully understood in α-synucleinopathy. Consequently, this review focuses on the pathogenic impact of α-synuclein phosphorylation and its kinases during the neurodegeneration process in α-synucleinopathy.

## 1. Introduction

With the increase in global aging, the number of patients with dementia and Parkinson’s disease is similarly growing. In Parkinson’s disease and dementia with Lewy bodies (DLB), the accumulation in the brain of a protein called α-synuclein is the pathological hallmark. α-Synuclein is a protein with 140 amino acids encoded by the *SNCA* gene. It is mainly expressed in the central nervous system in human adults. Most of the protein is distributed in the cytoplasm of cells; however, a portion is extracellularly detected, thus leading to postulation of its pathogenic ability. Aggregated α-synuclein is cytotoxic and plays a central role in the pathogenesis of synucleinopathies. Phosphorylation of α-synuclein presumably accelerates its fibrilization and the formation of insoluble aggregates. Several kinases catalyze phosphorylation; nonetheless, the role of phosphorylation enzymes in disease pathogenesis is currently not well understood. Therefore, in this review, we focus on the pathogenic impact of phosphorylation and its kinases during the neurodegeneration process in Parkinson’s disease.

## 2. Characteristics and Distribution of α-Synuclein

**Figure 1:**
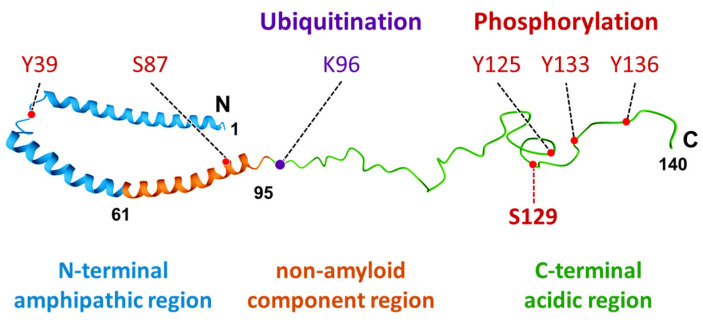
Structure and summary of post-translational modification sites of α-synuclein. The schematic represents α-synuclein protein [28] with the N-terminal amphipathic region, non-amyloid component region, and C-terminal acidic region. Phosphorylation sites are indicated in red letters, and ubiquitination is shown in purple. The indicated Ser129 phosphorylation site is the potential region mostly associated with α-synuclein-induced neurodegeneration.

α-Synuclein is a protein with a molecular weight of 14.5 kDa and consists of 140 amino acids encoded by the *SNCA* gene. The protein consists of three domains: the N-terminal amphipathic region, the non-amyloid component region, and the C-terminal acidic region (Figure 1). In the central nervous system, it is particularly abundant in presynaptic terminals and is presumed to be involved in regulating synaptic function and neural plasticity. Synuclein was first identified in cholinergic synaptic vesicles derived from the electric organ of the northeastern Pacific electric ray (*Torpedo californica*) [1]. The name “synuclein” originated from its distribution because its expression was observed not only in synaptic vesicles in the presynaptic terminal but also in the nucleus following immunostaining. Owing to this, α-synuclein was presumed to be a molecule related to the nervous system, especially in synaptic function.

α-Synuclein is particularly highly expressed in the central nervous system in human adults—accounting for approximately 1% of soluble proteins in the brain—and is abundant in presynaptic terminals [2]. α-Synuclein protein is also abundant in erythrocytes [3]. Most α-synuclein is distributed in the cytoplasm of cells and its carboxyterminal region, which is flexible in monomeric and fibrillar states and plays an essential role in the interaction with other partners [4]. Some of the protein is associated with biological membranes, such as synaptic vesicles [5]. In addition, α-synuclein is observed in the mitochondrial inner membrane, the mitochondria-associated endoplasmic reticulum membranes, the Golgi apparatus, and endosomes [6,7,8,9]. It also interacts with various proteins, including tau, ATPases, lipid membranes, lymphocyte-activation gene 3, flotillin, and fatty acid-binding protein 3 [10,11,12,13,14,15,16,17,18,19,20], as well as metal ions [21,22]. Nuclear α-synuclein presumably functions as a DNA-binding protein and is thought to be involved in regulating various gene expressions [23,24,25].

Although α-synuclein functions intracellularly, the protein is detected in small amounts in cerebrospinal fluid, serum, urine, and other bodily fluids [7,8,9]. It is also detectable in the culture medium supernatant of cultured neuronal cells [26,27], thus being extracellularly excreted in part. In addition, α-synuclein secretion is related to neuronal activity, with the released protein activating inflammatory responses in microglia [26,27]. Consequently, extracellular α-synuclein is of interest with regard to the prion-like propagation of Lewy body diseases.

## 3. Propagation of α-Synuclein and Neurodegeneration

Although the distribution of α-synuclein-positive Lewy bodies increases with the progression of Lewy body disease, cognitive and motor deficits are not observed in the early stages of α-synuclein accumulation, with the accumulation of the causative protein in the brain being significantly advanced when cognitive decline is observed. In 2003, Braak et al. proposed a model of lesion development in which α-synuclein appears in the dorsal medullary vagus nucleus in the early stages of the disease and then expands from the midbrain to the limbic system and neocortex in patients with Parkinson’s disease [29]. Furthermore, α-synuclein-positive Lewy-body-like inclusions were identified in neurons of fetal donor origin following an autopsy of brains affected by Parkinson’s disease that had undergone transplantation of fetal substantia nigra tissue fragments [30,31]. These reports suggest that α-synuclein may propagate from cell to cell and cause lesion expansion. Pathological studies have indicated that α-synuclein may appear in the peripheral nervous system, including the gastrointestinal mucosa and cardiac sympathetic neurons, and migrate to the central nervous system after 20 years [20,31,32,33,34].

Previous studies reproduced the cell-to-cell propagation of α-synuclein in several animal models. In A53T mutant α-synuclein-expressing mice, intracerebral administration of brain extracts from prion-treated old mice accelerated the pathology of α-synuclein and shortened survival time [35]. Furthermore, following the administration of human α-synuclein fibrils into the brains of wild-type mice, the administrated “seeds” were degraded and, in turn, undetectable, while endogenous mouse α-synuclein was phosphorylated, ubiquitinated, and accumulated three months after the administration [36]. In addition, injection of pathological α-synuclein preformed fibrils into the duodenal and pyloric muscular layers showed the spread of α-synuclein into the brain as determined by phosphorylation at serine 129 [31]. These data indicate that exogenous α-synuclein may serve as a template to facilitate the aggregation and phosphorylation of endogenous protein with propagation potential.

For the cell-to-cell propagation of α-synuclein, the uptake process into the cells is essential. Although the molecular mechanism by which α-synuclein is taken up into cells is still not fully understood, the involvement of various biomolecules has been reported in recent years. For example, Na^+^/K^+^-ATPase, a sodium-potassium exchange pump coupled with ATP hydrolysis, binds α-synuclein multimer via its extracellular domain [13]. In addition, the regulatory T-cell-associated protein LAG-317, neurexin 1b, and amyloid-β precursor-like protein 1 are possible receptors for α-synuclein [14]. α-Synuclein can also be internalized by the lipid raft-associated protein flotillin-1-dependent endocytosis of dopamine transporter [16]. Furthermore, fatty-acid-binding protein 3 coupled with dopamine D2 long-type receptor is critical for the uptake of α-synuclein, and knockout of these proteins abolishes α-synuclein accumulation and propagation [17,19,37]. These data indicate that various mechanisms participate in the internalization and accumulation of α-synuclein.

## 4. Kinases That Phosphorylate α-Synuclein and Their Association to Cytotoxicity

**Figure 2:**
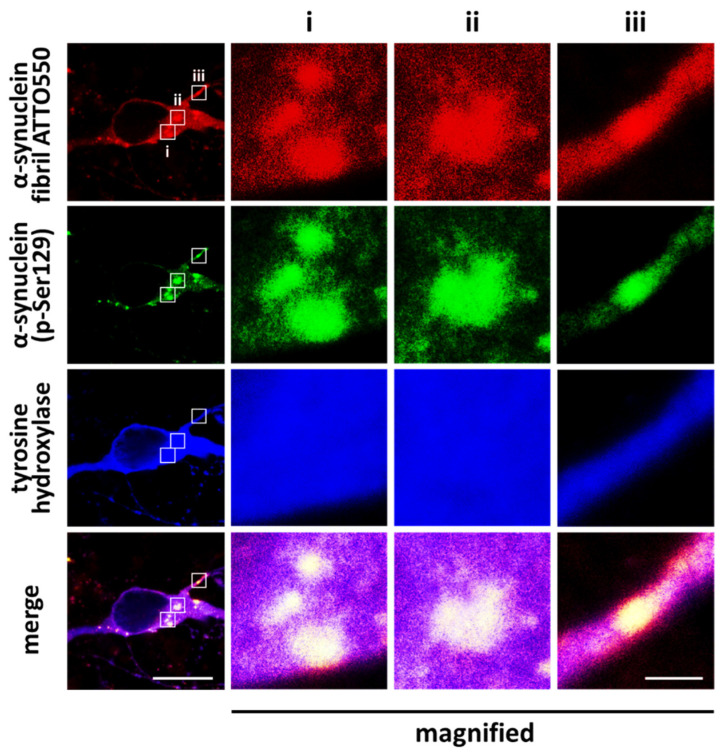
α-Synuclein Ser129 phosphorylation in cultured dopaminergic neurons. Exposure to exogenous α-synuclein fibrils induces the formation of phosphorylated α-synuclein-positive intracellular aggregates. Cells were exposed to ATTO550-labeled α-synuclein fibrils for 48 h. Scale bar = 10 μm, and 1 μm in magnified images of ROI i, ii, and iii. Images were originally obtained for this review.

Exogenous α-synuclein that is propagated and taken up into the cell facilitates aggregation with intracellular proteins to form inclusions, which are immunopositive for α-synuclein phosphorylated at Ser129 (Figure 2). Nonetheless, the question of how α-synuclein obtains these aggregation characteristics and enhances cytotoxicity arises. Earlier investigations reported that α-synuclein within Lewy bodies is subject to post-translational modifications, including phosphorylation and ubiquitination. Notably, among these modifications, the phosphorylation at residue Ser129 and its possible implication on α-synucleinopathy have had significant research focus and are postulated to play a key role in the regulation of α-synuclein aggregation and neurotoxicity [37,38,39]. In particular, a small portion of α-synuclein (~4%) is constitutively phosphorylated at Ser129 in the brains of healthy individuals [40,41,42], whereas >90% of the insoluble α-synuclein is phosphorylated in the brain of patients with synucleinopathies [41,43,44,45]. The accumulation of phosphorylated α-synuclein at Ser129 was also observed in animal models of Parkinson’s disease [46,47,48,49,50]. These observations indicate the importance of α-synuclein phosphorylation at Ser129 in regulating its aggregation and neurotoxicity.

**Figure 3:**
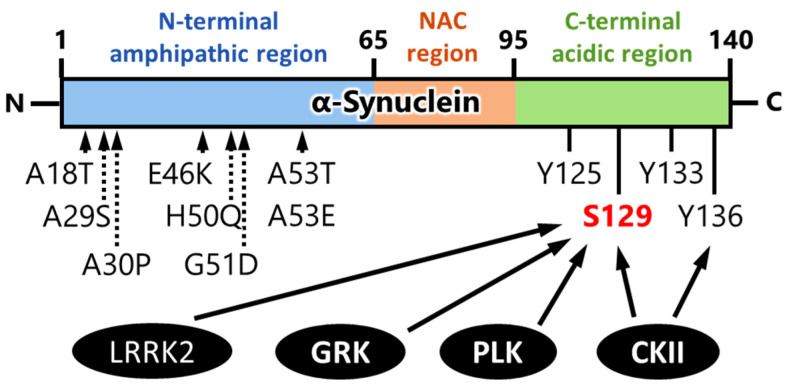
Summary of representative phosphorylation sites, kinases, and mutation sites of α-synuclein [54,55,56,57,58,59,60,61,62,63,64]. GRK: G-protein-coupled receptor kinase; CKII: casein kinase II; PLK: polo-like kinase; LRRK2: leucine-rich repeat kinase 2; NAC: non-amyloid component.

The carboxy-terminal region of α-synuclein is flexible in monomeric and fibrillar forms as well as in membrane-bound states [4,18,51,52,53]. Interestingly, the α-synuclein C-terminal tail contains the majority of phosphorylation sites (Figure 3). Phosphorylation of α-synuclein Ser129 is mediated by several kinases such as G-protein-coupled receptor kinases (GRKs) [54,55,56], casein kinase II [56,57,58], polo-like kinases [59,60,61], and leucine-rich repeat kinase 2 (LRRK2) [62,63]. Below, we summarize the α-synuclein phosphorylation kinases and describe the associated pathogenic neurotoxicity.

### 4.1. Impact of G-Protein-Coupled Receptor Kinases on α-Synuclein Phosphorylation

GRKs are a serine/threonine kinase family that regulate G-protein-coupled receptors. Mammalian GRKs have seven isoforms that can be divided into three subfamilies based on their structural organization and homology: GRK1 (rhodopsin kinase) and GRK7; GRK2 (βARK1) and GRK3 (βARK2); and GRK4, GRK5, and GRK6. Among these isoforms, GRK2 preferentially phosphorylates α-synuclein, while GRK5 prefers α-isoform as a substrate [54,55]. Interestingly, GRK-mediated authentic phosphorylation and mimicking of the phosphorylation state (S129E) reduce α-synuclein affinity to binding phospholipids [54,65]. Furthermore, phosphomimetic mutation (S129D) inhibits its association with membranes [66,67]. These data indicate an inhibitory effect of Ser129 phosphorylation on the α-synuclein binding to the cellular membrane. Following the loss of ability to bind to plasma membrane, phosphorylated α-synuclein preferentially localizes to mitochondria and suppresses cell respiration [68,69], which also induces endoplasmic reticulum stress and cell death [46,70,71]. These data indicate that α-synuclein phosphorylation is linked to mitochondrial dysfunction and the production of reactive oxygen species. GRK5 is immunohistochemically co-localized with phosphorylated α-synuclein in Lewy bodies [55], indicating its pathogenic significance in α-synucleinopathies.

### 4.2. Impact of Casein Kinase II on α-Synuclein Phosphorylation

Another kinase of α-synuclein phosphorylation, casein kinase II (CK2), is a serine/threonine-selective protein kinase. CK2 is a tetrameric enzyme assembled from two catalytic subunits (CK2α and CK2α′) and a regulatory subunit (CK2β dimer). Casein kinase II is necessary for cell survival and is a crucial suppressor of apoptosis [72]. Wu et al. reported that CK2α can be S-nitrosylated by nitric oxide (NO). Intriguingly, S-nitrosylation of CK2α enhanced the kinase activity of this catalytic subunit towards the phosphorylation of α-synuclein at Ser129 [56]. These data indicate that CK2α can enhance the phosphorylation of pSer129 α-synuclein. In addition, Sano et al. found that α-synuclein aggregates are predominantly phosphorylated at Y136 in brains with DLB, which is mediated by the activity of CK2 [58]. Furthermore, aggregate formation with Ser129 phosphorylation was significantly attenuated by CK2 inhibition, which increased Y136 phosphorylation in cultured neuroblastoma SH-SY5Y cells [58]. Moreover, CK2β regulatory subunits are detected in the halo region of Lewy bodies in the substantia nigra of patients with Parkinson’s disease [70]. These data indicate the involvement of CK2-related α-synuclein phosphorylation in the pathogenesis of Lewy-body-associated diseases.

### 4.3. Impact of Polo-like Kinase on α-Synuclein Phosphorylation

The third phosphorylation candidate, polo-like kinase, is also a regulatory serine/threonine kinase containing an N-terminal kinase catalytic domain and a C-terminal polo-box domain (PBD) that is involved in substrate binding and regulation of kinase activity. Five mammalian PLK family members have been identified, viz. PLK1–5 [71]. The polo-like kinases PLK2 and PLK3 have phosphorylation activity against α-synuclein that is better than that of PLK1 and PLK4 and are capable of completely phosphorylating α-synuclein [59,60]. PLK5 lacks a functional kinase domain due to a premature stop codon in exon 6 and is thus unable to phosphorylate α-synuclein. Interestingly, a significant reduction in α-synuclein phosphorylation has been observed in primary cortical neurons and the cerebral cortex of PLK2 knockout mice [59]. Bergeron et al. further demonstrated the ability of PLK2 to phosphorylate α-synuclein at Ser129 in the brain of mice in vivo [61]. These data show that polo-like kinase is critical for α-synuclein phosphorylation in the pathogenesis of α-synucleinopathy. Furthermore, dephosphorylated α-synuclein binding inhibits the activity of tyrosine hydroxylase (TH) [73], the rate-limiting enzyme in dopamine biosynthesis. Interestingly, phosphorylation of α-synuclein at Ser129 by PLK2 in vitro reduced the ability of α-synuclein to inhibit TH [74]. Active phosphorylated TH is easily aggregated [75,76] and degraded by the ubiquitin-proteasome system [77,78], indicating the attenuation of dopaminergic function. These data suggest the relevance of PLK2 in the reduced dopamine levels observed in Parkinson’s disease in an α-synuclein phosphorylation-dependent manner.

### 4.4. Impact of Leucine-Rich Repeat Kinase 2 on α-Synuclein Phosphorylation

Leucine-rich repeat kinase 2 (LRRK2) is a 286-kDa multi-domain containing a member of the receptor-interacting protein kinase (RIPK) family. LRRK2 consists of several domains, viz. N-terminal ankyrin repeats, leucine-rich repeats, a ras of complex (ROC) GTPase domain with an adjoining C-terminal of ROC (COR) domain, a serine/threonine-protein kinase domain, and C-terminal WD40 repeats. Mutations of the *LRRK2* gene were discovered to be the cause of familial Parkinson’s disease (PARK8) [79,80,81,82]. Interestingly, Qing et al. reported that LRRK2 has a significant capacity to phosphorylate recombinant α-synuclein at Ser129 [62,63]. Furthermore, Guerreiro et al. reported that the levels of LRRK2 are positively related to the increase in α-synuclein phosphorylation and aggregation in diseased brain regions, including the amygdala and anterior cingulate cortex [83]. The authors also reported the co-localization of these two proteins in the substantia nigra neuronal cells and Lewy bodies in the brain tissue of a patient with Parkinson’s disease [83]. Notably, LRRK2 and α-synuclein phosphorylated at Ser129 co-localized in a small proportion of the pathologies, and knockdown of LRRK2 expression did not produce significant changes in phosphorylated α-synuclein levels [83]. These data suggest the physiological significance of LRRK2 in the pathogenesis of Lewy-body-associated diseases. However, further investigations are required to reveal the relationship between LRRK2 and α-synuclein phosphorylation.

## 5. Physiological Significance of Phosphorylation on α-Synuclein Aggregation

As previously alluded, several kinases influence the phosphorylation of α-synuclein, and the status of the protein is evidently linked to the pathogenesis of Lewy body diseases. However, it is still ambiguous whether phosphorylation is the limiting step to promoting aggregation. In cell lines and primary neuronal cultures, exposure to the exogenous α-synuclein fibrils results in the formation of intracellular aggregation, which is immunopositive to α-synuclein phosphorylated at Ser129 [84,85] (Figure 2). However, phosphorylation does not seem to be mandatory for the formation of cytoplasmic inclusions. Luk et al. reported that phosphorylated α-synuclein was present only in the triton-insoluble fraction following biochemical analysis, suggesting this modification occurs to the growing inclusion after recruitment [85].

As a matter of fact, there is much debate about the effect of α-synuclein phosphorylation on protein aggregation. Previously, Fujiwara et al. reported that α-synuclein phosphorylated at Ser129 by CK2 was found to form fibrils more readily than dephosphorylated α-synuclein in vitro [41]. The genetic mutant to mimic Ser129 phosphorylation (S129D) was associated with pathology in a transgenic *Drosophila* model [49]. Furthermore, α-synuclein hyperphosphorylation and insolubility were correlated with the disease in transgenic mouse models of multiple system atrophy [86] and DLB [87]. Other investigations that made use of cell lines, primary cultures, and rat models further support the notion that α-synuclein phosphorylation promotes cytoplasmic inclusion formation in Parkinson’s disease [55,88,89,90,91]. Similar findings were also observed in a multiple system atrophy model using an oligodendroglial cell line [92]. These observations indicate that α-synuclein phosphorylation at Ser129 promotes its aggregation and inclusion formation in the pathogenesis of Lewy body diseases including Parkinson’s disease, DLB, and multiple system atrophy.

Controversially, other studies suggested that phosphorylation prevents or has no effect on inclusion formation in a *Drosophila* model [49] and in vitro [93,94]. In particular, phosphomimetic S129E mutation had no effect, while the dephosphomimetic mutation S129A increased α-synuclein toxicity in budding yeast [66,95]. Moreover, in rat models using retrovirus-mediated expression of α-synuclein in neurons of the substantia nigra, the S129A variant also showed toxicity. Contrarily, S129D showed protective [90] or no [96] effects. In a *Caenorhabditis elegans* model, S129D α-synuclein was also protective and reduced neuronal dysfunction, while S129A expression resulted in severe motor dysfunction and synaptic abnormality following reduced membrane interaction [67]. These data are apparently consistent with the α-synuclein characteristics that present soluble oligomeric or protofibrillar species as more toxic than larger aggregated forms. Furthermore, Schreurs et al. showed that Ser129 phosphorylation by PLK2 displays similar fibrillization kinetics to wild-type α-synuclein in vitro [97]. More recently, Weston et al. reported that aggregation of presynaptic α-synuclein is not influenced by its phosphorylation at Ser129 in vivo [98]. Collectively, these data demonstrate that phosphorylation is not a mandatory factor in the limiting step for α-synuclein aggregation as well as for enhancing pathogenic toxicity.

Several hypotheses may be proposed to explain the inconsistencies between the results obtained in different cell and animal models. One possibility is that phosphorylated protein is preferably ubiquitinated and degraded by the ubiquitin–proteasome system [78], which diminishes the phosphorylated soluble α-synuclein following its clearance in the aggregation process. α-Synuclein aggregation is not a single event and is affected by various modifications [99,100]. Further studies are required to understand the physiological significance of phosphorylation in the process of aggregation and formation of inclusions.

## 6. Pathological Impact of Phosphorylated Synuclein and Its Potential as a Novel Therapeutic Target

Previous investigations involving immunohistochemical and biochemical analyses have accumulated much data on Lewy bodies, showing the deposition of α-synuclein phosphorylated at Ser129 in postmortem brains of patients with Parkinson’s disease and DLB. Thus, it is worth investigating when the accumulation of phosphorylated protein begins in the various stages of Lewy body diseases. In this section, we summarize when phosphorylation at Ser129 occurs during the pathogenesis of Lewy body diseases and propose potential therapeutic targets to modulate α-synuclein phosphorylation.

Previously, Walker et al. reported that a biochemical analysis revealed progressive accumulation of p-Ser129 immunoreactivities as well as a positive correlation between phosphorylation level and the severity of symptoms of Parkinson’s disease and DLB [101]. Moreover, the accumulation of insoluble phosphorylated forms and the formation of p-Ser129-positive insoluble species became detectable only at the late stages of the diseases, stages IV and V, according to the Unified Staging System [101,102]. Zhou et al. also reported a dramatic accumulation of p-Ser129-positive inclusions in various brain regions at the late stages of the disease using postmortem brain samples from patients with Parkinson’s disease [103]. These findings revealed that pathogenic deposition of insoluble α-synuclein phosphorylated at Ser129 primarily occurs at the advanced stages of synucleinopathies.

Many kinases are activated in Parkinson’s disease and related disorders and are associated to the diseases’ pathogenesis. Focusing on the kinases that have the ability to phosphorylate α-synuclein (Figure 3), GRK, CK2, and PLK indeed alter the activity by their modification in Lewy body diseases. As introduced in Section 4, GRK6 and CK2α can be S-nitrosylated by NO, with S-nitrosylation enhancing their kinase activity towards the phosphorylation of α-synuclein at Ser129 [56]. Notably, in an A53T α-synuclein transgenic mouse, a familial Parkinson’s disease model, Wu et al. demonstrated that increased GRK6 and CK2α S-nitrosylation was age-dependent and associated with an increased level of p-Ser129 α-synuclein, which suggests GRK6 and CK2α S-nitrosylation as a potential therapeutic target of α-synucleinopathy. In fact, treatment of A53T α-synuclein transgenic mice with Nω-nitro-L-arginine (L-NNA), a NO synthase inhibitor, significantly reduced the S-nitrosylation of GRK6 and CK2α in the brain [56]. Additionally, Chau et al. reported that pramipexole (PPX) decreases the phosphorylation of CK2 to reduce its activity, thereby inhibiting the phosphorylation of α-synuclein at Ser129 [104]. Observation of LRRK2 phosphorylation by casein kinase 1α [105] also raises the possible development of another therapeutic agent.

**Figure 4:**
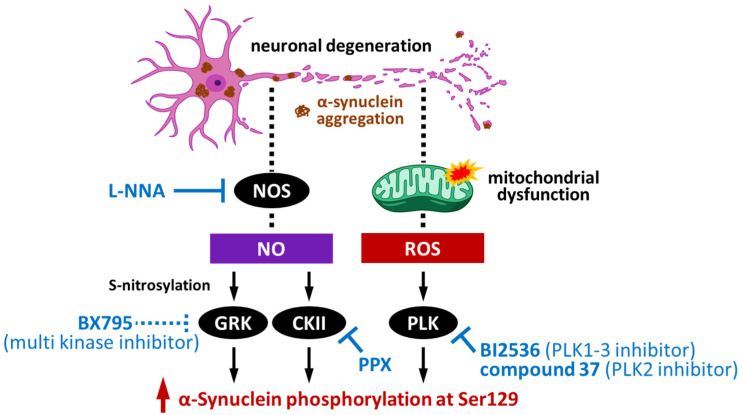
Schematic pathways of α-synuclein phosphorylation by kinases in the neurodegeneration process and potential pharmacotherapeutic targets [56,104,106,107,108,109,110]. NO: nitric oxide; NOS: NO synthase; ROS: reactive oxygen species; L-NNA: N(omega)-nitro-L-arginine, a competitive inhibitor of nitric oxide synthase; GRK: G-protein-coupled receptor kinase; CKII: casein kinase II; PLK: polo-like kinase; PPX: pramipexole.

PLK is also associated with the pathogenesis of Parkinson’s disease. Oxidative stress generated as a consequence of mitochondrial dysfunction can activate PLK2 [106]. Weston et al. demonstrated that PLK2 knockout reduces presynaptic terminal α-synuclein Ser129 phosphorylation [107]. In this condition, PLK2 deletion has no effect on the phosphorylation levels in Lewy-body-like inclusions [107], suggesting that the formation of inclusions is α-synuclein-phosphorylation-independent. Furthermore, PLK2 deletion slows the rate of neuronal death [107], indicating that inhibition of PLK2 is a potential therapeutic target. Ingris et al. demonstrated that treatment with the PLK inhibitor BI2536 inhibited α-synuclein phosphorylation at Ser129 [59]. Furthermore, treatment with a designed highly selective inhibitor for PLK2, compound 37, reduced α-synuclein phosphorylation in rat brain [108,109]. However, the inhibitor had no effect on α-synuclein aggregation, p-Ser129, or inter-neuronal spreading of the aggregated α-synuclein in the S129A preformed fibril injection model [110]. These findings suggest that PLK2 could be a potential target for the development of novel therapies, although further optimization is still required at the current stage (Figure 4).

In recent years, the detection of phosphorylated α-synuclein at Ser129 in human cerebral spinal fluid and blood plasma has been highlighted as a viable biomarker for diagnosis of α-synucleinopathies [111]. The result of the concentration of total α-synuclein in the plasma reportedly varied, with some work showing no difference between healthy controls and diseased patients [112] and others showing lower [113,114] or higher levels [115,116,117]. However, p-Ser129 α-synuclein levels were significantly higher in samples from patients with Parkinson’s disease [111,112]. In addition, the analysis of skin biopsies revealed that the majority of patients with Parkinson’s disease show an accumulation of p-Ser129 α-synuclein, while no immunoreactivity was detected in healthy controls [118,119]. Furthermore, other studies reported the presence of p-Ser129-immunoreactivities in gastric, duodenal, and colonic biopsies [120,121]. These observations suggest that α-synuclein phosphorylation is not only physiologically crucial in the pathogenesis of Lewy body diseases but is also a potential biomarker for the diagnosis and monitoring of the progression of Parkinson’s disease and related disorders.

## 7. Conclusions

This review summarizes the physiological significance of α-synuclein-phosphorylating kinases and the impact of α-synuclein phosphorylation in the pathogenesis of α-synucleinopathies. α-Synuclein phosphorylation can clinically be an accompanying event in the brains of patients with Parkinson’s disease rather than the critical factor for α-synuclein aggregation and toxicity. Nevertheless, increasing phosphorylated α-synuclein and the accumulation with disease progression is useful as a therapeutic target and biomarker. We recently showed that clearance of p-Ser129 α-synuclein alleviates cognitive and motor dysfunction in a Parkinson’s disease mouse model [50]. We also developed a therapeutic compound targeting metal ions, which has the potential to modulate α-synuclein metal binding and alleviate its accumulation [122,123]. Based on our finding that fatty-acid-binding proteins are critical for the accumulation of α-synuclein following phosphorylation [17,19,37], we intend to develop more fundamental therapeutics and diagnostic tools for α-synucleinopathies, including Parkinson’s disease, DLB, and multiple system atrophies.

## Data Availability

Not applicable.
